# A Few Thoughts, Etc.

**Published:** 1853-08

**Authors:** Chas. L. Anderson

**Affiliations:** St. Anthony Falls, Minnesota


*A Few Thoughts on Periodicity in Disease.*—By Chas. L. Anderson, M. D., St. Anthony Falls, Minnesota.

> "Through all thy veins shall run
> A cold and drowsy humor, which shall seize
> Each vital spirit."—Shakspeare.

A physician meets with more things in his practice than all the philosophies have ever "dreamt of." And many a time is his poor brain tortured to find a *cause* or a *reason* why. A lawyer may make his cases bend to the established "precedent," and to the rules of Coke, Kent, or Blackstone. A minister has his established creed, and each week, with sermon neatly arranged under "three heads," he expounds it to his somnolent hearers. But the physician finds that "precedents" and "rules," and an arrangement under the "three heads" will not always answer his anticipations. He must be a physical observer, an analogical reasoner, and an expectant of anomalous cases. He cannot follow a one-idea course with justice to his patients. He must not be ultra nor rigid, and yet he *must* be decided. It is true he has facts before him from which to draw his inferences; but even these may lead him to a false conclusion, unless he take into the account other facts of, frequently, an antagonistic nature.

In the few thoughts I propose to offer on the subject of Periodicity in Disease, my aim will be to arrive at practical conclusions, instead of establishing any vague theories. I will also endeavor to be as simple and concise as the intricacy of my subject will possibly permit.

Periodic motion is characteristic of things, whether ponderable or imponderable. It is apparently inherent to all forces resident in matter, as well as to the masses of matter. A few familiar examples will illustrate this. A body cast into the air will cease to ascend as soon as the impulse which it received is overcome by the resistance of the atmosphere, and by what we term gravity. It then descends, inversely, with the same velocity as it ascended. The vibrations of the pendulum afford another example of periodic motion. The ripples that surround a pebble when cast into a quiet lake—the magnetic battery—the vibratory nature of sounds—in short, all motion must be periodic; although one kind may be more perceptible than another.

Of the actions going on around us, we recognise two as most prominent, viz: Organization and Disorganization—Growth and Decay—Life and Death. And these are subject to that omniprevalent law, Periodicity.

From the mineral the vegetable grows, and may from thence be assimilated to the animal. But this is the acme, or extreme of the vibration. Reaction ensues; it is again returned to dust, prepared for another impulse and another reaction.

Thus, briefly, we observe the nature of Periodicity, its prevalence, and its variations. Of its object it matters little to my subject. I will merely state as a proposition, that the tendency of every motion, after the impulsive force ceases to act, is to that of an original condition; or rather, to an equilibrium. To illustrate,—the pendulum seeks its starting point—the disturbed wave seeks rest—the "fitful gusto" of wind endeavors to preserve a balance—an increased action is followed by a proportionate diminution, and then a gradual restoration. This primitive tendency appears to be the grand object of motion; and from our idea of motion it is impossible to separate the idea of periodicity.

Life and motion are so intimately connected that we scarcely know how to define a difference. So much so that an eminent poet and student of nature has written:—

"From the star
To the winding worm, all life is motion;
And in life, *commotion* is the extremest point
Of life. The planet wheels till it becomes
A comet, and, destroying as it sweeps
The stars, goes out. The poor worm winds it way
Living upon the death of other things;
But still, like them, must live and die the subject
Of something which has made it live and die.

BYRON.

Having endeavored to present to the mind a few palpable examples of the general aspects of periodicity as observed in all of Nature's works,—(for the physician must consult Nature and follow her teachings if he hopes for success in his practice) I shall now speak of the nature of those diseases which are eminently characterized by periodic action.

The first and most prominent is the common Intermittent Fever. In this, regular paroxysms occur, with scarce an hour's deviation from a fixed time; and the paroxysms are repeated almost any number of times without a second application of the cause. Now what is the nature and pathology of this disease? We must appeal first to the morbid anatomy as observable in *post mortem* examinations; and secondly, to the symptoms manifested during its progress. The most certain, and indeed almost the only appearance, in a *post-mortem*, is that of congestion, and its usual consequences. The brain, liver, lungs, and spleen seem to suffer most,—the latter organ almost invariably. We are led from a careful study of this disease to regard it as the result of a disturbance in the nervous equilibrium in the first place, and resulting in a depravation of the blood and derangement of the absorbent and secretory functions. Malaria may be, and is most commonly, the cause of this disturbance—but other causes may produce a similar effect. This disease, then, being primarily, and as some *post-mortems* show, entirely a difficulty in the nervous functions, we are led to examine the nature of nervous diseases. Even in health, the nervous phenomena evince a periodic character. Sleep, appetite, cultivated habits, and many other things, afford common examples. But in the case of an ague, the nervous force being for some time impeded by an antagonistic force, at last rallies to expel, as it were, a poison which had formed, and is being formed, (perchance by some unknown chemical affinities,) in the system; and the chill, so often observed, is the consequence. It is an effort of Nature to effect a cure. But the powerful force and congestion in the suffering organs does not always leave the individual unharmed. Lesions of the brain, lungs, liver, spleen, &c., may result. But why does another paroxysm return in a certain length of time? I answer, merely because of the disturbed equilibrium; or a re-application of the first cause, until the nervous system has acquired, as it were, a *habit*. To illustrate by the example of the pendulum, I might say that the chill and internal congestion is one extreme in the vibratory action, and that the consequent fever and external heat is the other extreme. The impulse has been given by the introduction of an agent which acts chemically upon the vital forces. And this impulse being once given, and the vibration commenced, it is as natural for a second paroxysm to occur as it is for the pendulum to vibrate a second time.

I need scarcely point out the practical inference that would arise from the above reasoning. The natural mode of treatment would be to equalize the disturbance in the circulatory, secretory, and nervous organs. Means for this are ample and have long since been resorted to with notorious success by physicians as well as empirics. But for *what reason*, I aver, few have ever hesitated to think; except that experience had shown such a mode to be most successful.

A word in regard to *critical days*, and I close this article. From the periodic character of disease arose the idea of critical days. The ancient Egyptians, it is said, were able to foretell the day and hour of a patient's death. Ætius, for this purpose, laid down rules, founded upon the crisis in disease. Whether these rules are practicl is extremely doubtful. But that there are regular and fixed periods, or crises, in every disease, I have no doubt. But when we come to consider that each case presents new combinations—not like any other that has ever occurred before it, or ever will occur after it,—we conclude that it is beyond "mortal ken" at present to prognosticate with certainty as to the future events of any case of disease.

But here is a field for philosophical investigation, that may be investigated with great value to the science of Medicine.

